# Association Between Body Mass Index and the Efficacy of Calcium Channel Blockers for Hypertension in Cardiovascular Disease Patients

**DOI:** 10.7759/cureus.81985

**Published:** 2025-04-10

**Authors:** Bassel Abdallah, Ahmed Jamal Chaudhary, Muhammad Waqas Javed, Marium Nadeem Khan, Ayesha Bibi, Muhammad Fayyaz Zafar, Muhammad Noor, Usman Tariq, Farzana Salman

**Affiliations:** 1 Department of General Internal Medicine, Royal Alexandra Hospital, Glasgow, GBR; 2 Department of Internal Medicine, Detroit Medical Center (DMC) Sinai-Grace Hospital, Michigan State University, Detroit, USA; 3 Department of Medicine, Shifa International Hospital, Shifa College of Medicine, Islamabad, PAK; 4 Department of Cardiology, Mayo Hospital Lahore, Lahore, PAK; 5 Department of Adult Critical Care, Sindh Infectious Diseases Hospital, Karachi, PAK; 6 Department of General Medicine, Countess of Chester Hospital NHS Foundation Trust, Chester, GBR; 7 Department of Internal Medicine, The First Affiliated Hospital of Changsha Medical University, Changsha Medical University, Changsha, CHN; 8 Department of Physiology, Peshawar Medical College, Peshawar, PAK

**Keywords:** blood pressure management, body mass index, calcium channel blockers, cardiovascular diseases, hypertension

## Abstract

Background: Hypertension, or high blood pressure, is a major risk factor for cardiovascular diseases (CVDs) worldwide. Variations in body mass index (BMI) may influence the efficacy of calcium channel blockers (CCBs) by affecting drug metabolism, vascular resistance, and inflammatory responses associated with adipose tissue.

Objective: This study aims to evaluate the association between BMI and the short-term efficacy of CCBs in managing hypertension among patients with CVDs over a six-month follow-up period.

Methodology: This prospective observational study was conducted at the Department of General Internal Medicine, Royal Alexandra Hospital, Glasgow, UK, from June 2023 to June 2024, enrolling 220 patients diagnosed with hypertension and at least one underlying cardiovascular condition, such as coronary artery disease, heart failure, or atrial fibrillation. Patients were categorized into BMI groups based on the World Health Organization (WHO) classification, and all were prescribed CCBs either as monotherapy or in combination with other antihypertensive medications. Blood pressure was measured using an automated sphygmomanometer with follow-up ambulatory monitoring, while lipid levels were assessed via fasting blood samples.

Results: The study involved 220 participants, categorized into four BMI groups: underweight (n = 40), normal weight (n = 60), overweight (n = 60), and obese (n = 60). Underweight patients had a baseline systolic/diastolic blood pressure of 150/95 mmHg, which decreased to 135/85 mmHg, showing a reduction of 15/10 mmHg. Normal weight patients experienced a drop from 145/90 mmHg to 130/80 mmHg, overweight patients from 155/95 mmHg to 140/85 mmHg, and obese patients from 160/100 mmHg to 145/90 mmHg, all with the same reduction of 15 mmHg in systolic and 10 mmHg in diastolic pressure. Low-density lipoprotein (LDL) levels decreased in all groups, with a reduction of 5 mg/dL in the underweight (130 to 125 mg/dL) and normal weight (125 to 120 mg/dL) groups, while the overweight (140 to 130 mg/dL) and obese (150 to 140 mg/dL) groups showed a greater reduction of 10 mg/dL. High-density lipoprotein (HDL) levels improved in all categories, increasing by 5 mg/dL in each group. LDL reduction was more pronounced in overweight and obese groups, likely due to metabolic changes associated with higher body fat. Adverse effects, including peripheral edema and dizziness, were more common in higher BMI groups, with a noticeable decline in medication adherence in obese patients. These results suggest that BMI may influence treatment efficacy, particularly in lipid regulation and the occurrence of adverse effects.

Conclusion: BMI does not significantly affect the blood pressure-lowering efficacy of CCBs in patients with hypertension and CVDs. However, a greater reduction in LDL levels was observed in overweight and obese groups, suggesting that BMI may influence lipid metabolism differently than blood pressure regulation.

## Introduction

Hypertension is a leading risk factor for cardiovascular diseases (CVDs) and often coexists with conditions such as heart failure, coronary artery disease, and atrial fibrillation, collectively contributing to increased morbidity and mortality [1]. Calcium channel blockers (CCBs), including amlodipine, diltiazem, and verapamil, are commonly prescribed antihypertensive agents due to their vasodilatory effects, heart rate reduction, and decreased cardiac workload [2,3]. However, treatment response to CCBs is highly variable, with approximately 10-30% of hypertensive patients classified as non-responders, depending on underlying comorbidities and demographic factors [4,5]. This variability highlights the importance of identifying factors that may influence drug efficacy, particularly body mass index (BMI), which categorizes individuals as underweight, normal weight, overweight, or obese. Given the rising global prevalence of obesity, investigating its impact on CCB responsiveness is both timely and essential [6].

Obesity (BMI ≥ 30 kg/m²) is a well-established contributor to hypertension through several mechanisms, including increased sympathetic nervous system activity, insulin resistance, and activation of the renin-angiotensin-aldosterone system (RAAS). These processes contribute to vascular remodeling and endothelial dysfunction, thereby altering the hemodynamic profile of patients [6-9]. Such changes may also affect the pharmacokinetics and pharmacodynamics of CCBs, influencing drug absorption, metabolism, and distribution across tissues [3,6,8,9]. Additionally, obesity-associated vascular changes, such as increased vascular smooth muscle mass, arterial stiffness, and reduced nitric oxide (NO) bioavailability, can significantly modulate the vasodilatory effects of CCBs [10].

Evidence suggests that enhanced vascular resistance in obese individuals may blunt CCB-induced vasodilation, reducing therapeutic efficacy [11]. Conversely, altered calcium signaling in hypertrophic vascular smooth muscle may, in some cases, increase sensitivity to CCBs, making the response pattern somewhat unpredictable [12]. These conflicting effects point to the need for better stratification of hypertensive patients based on obesity-related vascular phenotypes.

While chronic low-grade inflammation is a hallmark of obesity, its direct role in modifying CCB response is still under investigation. Inflammatory mediators may influence vascular reactivity, calcium channel expression, and drug metabolism, potentially via cytochrome P450 enzyme pathways [13-15]. Though some systematic reviews have highlighted the link between obesity-related inflammation and cardiovascular risk, few have focused specifically on how these pathways influence antihypertensive drug efficacy, a gap that warrants further research.

Despite the well-known relationship between obesity and hypertension, relatively few studies have directly assessed how BMI influences CCB treatment outcomes. Existing data suggest that overweight and obese patients may have reduced antihypertensive responses, possibly due to altered drug distribution, vascular stiffness, or metabolic dysfunction [16]. Other metabolic factors, such as insulin resistance, physical inactivity, dyslipidemia, and medication adherence, may further complicate this relationship. While prior research has occasionally considered these as confounders, they are often not systematically controlled, limiting the ability to draw definitive conclusions [17].

Given these complexities, personalized treatment strategies may be necessary to optimize blood pressure (BP) control in obese hypertensive patients. Some clinical evidence suggests that higher doses of CCBs or combination therapy with agents such as beta-blockers or diuretics may be more effective in this population [18,19]. However, definitive recommendations remain elusive due to a lack of large-scale pharmacokinetic and pharmacodynamic studies focused on BMI-stratified dosing. In the context of poor BP control, obese individuals face a heightened risk of adverse cardiovascular outcomes, including stroke, myocardial infarction, and heart failure, underscoring the urgency of refining therapeutic approaches for this high-risk group [20-22].

The primary objective of this study is to evaluate the association between BMI and the short-term efficacy of CCBs in the management of hypertension among patients with CVDs over a six-month follow-up period. Specifically, the study investigates how different BMI categories (normal weight, overweight, and obese) affect the degree of BP reduction achieved with CCB therapy. In addition, the study explores secondary outcomes, including the incidence of CCB-related side effects, patterns of medication adherence, and changes in renal function, to provide a more comprehensive understanding of how BMI may influence both the therapeutic effectiveness and tolerability of CCBs.

## Materials and methods

Study design and setting

This study employed a prospective cohort design conducted at Shifa International Hospital Ltd., Islamabad, Pakistan, for patient recruitment and primary data collection, spanning from June 2023 to June 2024, with a six-month follow-up period. Ethical approvals were obtained, and data and biological samples were subsequently transferred to the Department of General Internal Medicine, Royal Alexandra Hospital, Glasgow, UK, for further analysis.

Participants

Inclusion Criteria

Adult patients aged 40 to 75 years with a documented diagnosis of essential hypertension (systolic BP ≥ 140 mmHg or diastolic BP ≥ 90 mmHg at screening) and at least one of the following comorbid cardiovascular conditions were eligible: heart failure (New York Heart Association class I-III) [23], stable coronary artery disease (defined as prior myocardial infarction, stable angina, or prior coronary revascularization more than three months prior to enrollment), or documented atrial fibrillation or flutter. Participants were required to be on a stable regimen of CCBs for at least three months prior to enrollment, either as monotherapy (amlodipine, nifedipine, diltiazem, or verapamil) or in combination with other antihypertensive agents (angiotensin-converting enzyme (ACE) inhibitors, angiotensin receptor blockers, beta-blockers, or diuretics). All participants provided written informed consent.

Exclusion Criteria

Patients with secondary hypertension (e.g., due to hyperaldosteronism, renal artery stenosis, or pheochromocytoma), pregnant or breastfeeding women, individuals with significant hepatic impairment (Child-Pugh class B or C), significant renal impairment (estimated glomerular filtration rate (eGFR) <30 mL/min/1.73 m², calculated using the Chronic Kidney Disease Epidemiology Collaboration (CKD-EPI) formula), known allergy or contraindication to any CCB, or those unable or unwilling to comply with the study protocol were excluded. Patients who had experienced a major cardiovascular event (e.g., myocardial infarction, stroke, or hospitalization for heart failure) within the three months preceding enrollment were also excluded to ensure a stable baseline.

Sample size and sampling method

This study utilized a non-probability consecutive sampling method. All eligible patients presenting to the cardiology and general medicine clinics at Shifa International Hospital Ltd. during the recruitment period (June 2023 to June 2024) were screened based on electronic medical records and invited to participate. A total of 220 patients meeting the inclusion criteria and providing informed consent were enrolled. Due to the exploratory nature of this initial study and resource constraints, a formal sample size calculation was not performed. The aim was to capture a broad representation of the target population within the defined timeframe.

Data collection

Upon enrollment, a comprehensive baseline assessment was conducted. This included the detailed collection of demographic information such as age, gender, and ethnicity, as well as smoking status, alcohol consumption, and a thorough past medical history, specifically focusing on the duration and management of hypertension and any comorbid cardiovascular conditions. A physical examination was performed, during which height and weight were measured using standardized equipment to calculate the BMI. Based on their BMI, patients were categorized into underweight (BMI < 18.5), normal weight (BMI = 18.5-24.9), overweight (BMI = 25.0-29.9), and obese (BMI ≥ 30.0). Heart rate was also measured after a five-minute rest period. Blood pressure was assessed by taking three consecutive readings in a seated position following a five-minute rest, utilizing a calibrated automated sphygmomanometer (Omron HEM-9200T, Omron Healthcare, Kyoto, Japan) on the non-dominant arm, unless medically contraindicated. The average of the final two readings was recorded for subsequent analysis. The same model of sphygmomanometer was consistently used for all baseline and follow-up blood pressure measurements conducted at Shifa International Hospital. Furthermore, a standard 12-lead electrocardiogram (ECG) was performed and interpreted by a qualified cardiologist. Baseline laboratory investigations involved the collection of venous blood samples after an overnight fast, which were processed within two hours at Shifa International Hospital's central laboratory. These measurements included a lipid profile, encompassing total cholesterol, low-density lipoprotein (LDL) cholesterol, high-density lipoprotein (HDL) cholesterol, and triglycerides, all analyzed using a Siemens ADVIA 2400 Chemistry System (Siemens Healthineers, Erlangen, Germany) with standard enzymatic assays. Renal function was evaluated through the measurement of serum creatinine and blood urea nitrogen (BUN) using the same Siemens ADVIA 2400 Chemistry System, and the eGFR was calculated using the CKD-EPI equation. A detailed medication history was also obtained, documenting all current medications, including the specific type and dosage of the CCB, any other antihypertensive agents, and medications being taken for existing comorbidities. Finally, lifestyle factors were assessed through structured interviews conducted by trained research staff, gathering information on physical activity levels using a modified version of the International Physical Activity Questionnaire Short Form, dietary habits via a brief food frequency questionnaire, and patterns of smoking and alcohol consumption (see Appendices).

Data management and quality control

Data were recorded on standardized case report forms (CRFs) at Shifa International Hospital by trained research staff. Double data entry was performed, and inconsistencies were resolved by cross-referencing with the original CRFs. Data were stored in a secure, password-protected database. All biological samples were labeled with unique identifiers to ensure anonymity. Data transfer between sites was conducted using secure, encrypted methods, adhering to relevant data protection regulations.

Sample handling and transfer

Following the baseline assessment, biological samples, specifically serum and plasma, were carefully aliquoted and stored at a temperature of -80°C within the central laboratory of Shifa International Hospital. For subsequent analysis in the United Kingdom, these samples were transported to the Department of General Internal Medicine at the Royal Alexandra Hospital in Glasgow using temperature-controlled dry shippers equipped with continuous temperature monitoring to ensure the maintenance of sample integrity throughout the journey. Upon arrival at the Royal Alexandra Hospital, the samples were immediately stored at -80°C until the time of analysis, which was performed within four weeks of their arrival.

Follow-up assessments

During the six-month study period, patients returned for follow-up assessments every two months at Shifa International Hospital Ltd., Pakistan. At each visit, blood pressure was re-measured following the same standardized protocol and using the identical Omron HEM-9200T automated sphygmomanometer employed at baseline. Patients were systematically queried about the occurrence of any new or worsening side effects that they perceived to be related to their CCB medication, using a standardized questionnaire to ensure consistency in reporting. Medication adherence was comprehensively assessed through a combination of methods: firstly, by conducting a pill count, where patients were asked to bring all their current medications to the clinic, and the remaining pills were counted by the research staff to calculate the proportion of medication taken since the previous visit; and secondly, through the administration of the eight-item Morisky Medication Adherence Scale (MMAS-8) [24], a self-report questionnaire designed to evaluate various aspects of medication-taking behavior. Any changes in the patients' lifestyle factors, particularly their levels of physical activity and dietary habits, since the preceding visit were recorded using the same questionnaires that were administered at the baseline assessment. Patient weight was also measured at each follow-up visit using the same standardized scale that was used initially. At the conclusion of the six-month follow-up period, repeat laboratory investigations were conducted, involving the measurement of the lipid profile (total cholesterol, LDL cholesterol, HDL cholesterol, triglycerides) and renal function tests (serum creatinine, BUN, eGFR) using the same Siemens ADVIA 2400 Chemistry System within Shifa International Hospital's central laboratory to maintain consistency with baseline measurements.

Medication dosage adjustments

At the initiation of the study, all participants continued on the dose of their CCB that they were prescribed prior to enrollment. During the follow-up visits, which occurred every two months, the treating physicians at Shifa International Hospital had the discretion to make adjustments to the dosage of the CCB based on their clinical evaluation of the patient's response to treatment and their adherence to established hypertension management guidelines, such as those published by the European Society of Cardiology/European Society of Hypertension (ESC/ESH) [25]. Any changes made to the medication dosage were carefully documented in the patient's individual study record. The most frequently prescribed CCB in the study cohort was amlodipine, and typical dosage adjustments observed ranged from a starting dose of 5 mg once daily, which could be titrated upwards to 10 mg once daily or, in some cases of side effects such as edema or hypotension, down-titrated as deemed clinically appropriate by the treating physician.

Outcome measures

The primary outcome of this study was the absolute change observed in both systolic and diastolic blood pressure measurements from the initial baseline assessment to the final six-month follow-up visit. In addition to this primary endpoint, several secondary outcomes were also evaluated. These included the incidence and severity of any reported side effects, analyzed across the different BMI categories defined in the study. We also assessed the differences in medication adherence rates among the various BMI categories, utilizing data obtained from both the pill counts and the scores on the MMAS-8. Furthermore, changes in the lipid profile, specifically levels of LDL cholesterol, HDL cholesterol, and triglycerides, from the baseline measurements to the six-month follow-up were examined in relation to BMI categories. Similarly, changes in markers of renal function, including serum creatinine and the eGFR, over the same six-month period were analyzed across the BMI groups. Finally, the study also tracked the time elapsed until a modification of the patient's antihypertensive medication regimen was required, either through an adjustment in the dosage of the CCB or the addition of other antihypertensive agents, due to either inadequate control of blood pressure or the occurrence of intolerable side effects.

Statistical analysis

Data analysis was performed using SPSS version 27 (IBM Corp., Armonk, NY, USA). Descriptive statistics (means, standard deviations, frequencies, and percentages) were used to summarize baseline characteristics of the study population and across BMI categories. Chi-square tests or Fisher's exact tests were used to compare categorical variables, and independent samples t-tests or one-way ANOVA were used to compare continuous variables between BMI groups at baseline.

Longitudinal changes in blood pressure, lipid profiles, and renal function within each BMI group were assessed using paired t-tests or repeated measures ANOVA. Multivariate linear regression models were constructed to assess the independent effect of BMI on the change in systolic and diastolic blood pressure at six months, adjusting for potential confounders. Confounders, including age, gender, baseline systolic and diastolic blood pressure, baseline comorbidities (e.g., diabetes and chronic kidney disease), the number of other antihypertensive medications at baseline, baseline lifestyle factors (physical activity level, dietary habits), and changes in these lifestyle factors during the study, as well as medication adherence (using the continuous score from the MMAS-8), were selected based on clinical relevance and prior research in hypertension treatment [20,26], which identified these factors as significant predictors of treatment outcomes. The specific variables included in each regression model will be detailed in the results section.

Logistic regression analysis was used to identify predictors of inadequate blood pressure control at six months (defined as systolic BP ≥ 140 mmHg or diastolic BP ≥ 90 mmHg), with BMI as a primary predictor of interest, adjusting for the same potential confounders as in the linear regression models. Kaplan-Meier survival analysis was pre-specified in the study protocol [27] to estimate the time to medication dosage adjustment or addition of other antihypertensive agents due to adverse effects, with BMI as a stratifying variable, and the log-rank test was used to compare survival curves between BMI groups. A p-value of <0.05 was considered statistically significant for all analyses, and 95% confidence intervals (CIs) were reported where applicable.

Ethical considerations

The study protocol was approved by the ethical review boards of Shifa International Hospital Ltd., Islamabad, Pakistan, and the NHS Greater Glasgow and Clyde Research and Development Department, UK. The study was conducted in accordance with the principles of the Declaration of Helsinki. All participants provided written informed consent prior to enrollment.

## Results

The study included a total of 220 patients with a mean age of 62.4 ± 8.7 years, with a male predominance (130, 59.1%) and 90 (40.9%) females. The average BMI was 26.9 ± 5.2 kg/m², with 40 (18.2%) classified as underweight, 60 (27.3%) as normal weight, 60 (27.3%) as overweight, and 60 (27.3%) as obese. While there was a higher representation of overweight and obese patients, this distribution should be considered when generalizing the findings to populations with a different BMI distribution. The mean duration of hypertension was 8.2 ± 3.5 years. Among the participants, 110 (50.0%) had coronary artery disease, 75 (34.1%) had heart failure, and 60 (27.3%) had atrial fibrillation. Additionally, 95 (43.2%) patients had diabetes mellitus, while 120 (54.5%) had dyslipidemia. The distribution of comorbidities was noted across BMI categories, and it was observed that these metabolic conditions may influence blood pressure responses. A total of 40 (18.2%) patients were current smokers. These findings (Table 1) highlight the high burden of cardiovascular comorbidities in hypertensive patients, underscoring the need for personalized treatment approaches considering both BMI variations and associated risk factors.

**Table 1:** Demographic characteristics of study participants. The "±" symbol represents the mean and standard deviation (SD) in statistical analysis. The mean indicates the average value, while the standard deviation measures the variability or dispersion of the data around the mean.

Variable	Value
Total patients	220
Age (years)	62.4 ± 8.7
Male (%)	130 (59.1%)
Female (%)	90 (40.9%)
BMI (kg/m²)	26.9 ± 5.2
Underweight (%)	40 (18.2%)
Normal weight (%)	60 (27.3%)
Overweight (%)	60 (27.3%)
Obese (%)	60 (27.3%)
Hypertension duration (years)	8.2 ± 3.5
Coronary artery disease (%)	110 (50.0%)
Heart failure (%)	75 (34.1%)
Atrial fibrillation (%)	60 (27.3%)
Diabetes mellitus (%)	95 (43.2%)
Dyslipidemia (%)	120 (54.5%)
Current smoker (%)	40 (18.2%)

The study involved 220 participants, categorized into four BMI groups: underweight (n = 40), normal weight (n = 60), overweight (n = 60), and obese (n = 60). Underweight patients had a baseline systolic/diastolic blood pressure of 150/95 mmHg, which decreased to 135/85 mmHg, showing a reduction of 15/10 mmHg. Normal weight patients experienced a drop from 145/90 mmHg to 130/80 mmHg, overweight patients from 155/95 mmHg to 140/85 mmHg, and obese patients from 160/100 mmHg to 145/90 mmHg, all with the same reduction of 15 mmHg in systolic and 10 mmHg in diastolic pressure (Table 2).

**Table 2:** Blood pressure reduction data. F = ANOVA test. P < 0.05 was significant. SBP: systolic blood pressure; DBP: diastolic blood pressure.

BMI group (N)	Baseline SBP (mmHg) ± SD	Follow-up SBP (mmHg) ± SD	SBP reduction (mmHg) ± SD	Baseline DBP (mmHg) ± SD	Follow-up DBP (mmHg) ± SD	DBP reduction (mmHg) ± SD	F-value	P-value (SBP reduction)	F-value	P-value (DBP reduction)
Underweight (40)	150 ± 12	135 ± 10	15 ± 5	95 ± 8	85 ± 7	10 ± 4	0.56	0.64	0.42	0.74
Normal weight (60)	145 ± 11	130 ± 9	15 ± 4	90 ± 7	80 ± 6	10 ± 3				
Overweight (60)	155 ± 13	140 ± 11	15 ± 5	95 ± 9	85 ± 8	10 ± 4				
Obese (60)	160 ± 14	145 ± 12	15 ± 6	100 ± 10	90 ± 9	10 ± 5				

Table 3 shows that BMI remained stable in the underweight and normal weight groups, with no significant change from their baseline values of 18.0 kg/m² and 22.0 kg/m², respectively. In contrast, the overweight group experienced a slight reduction in BMI from 27.5 kg/m² to 26.8 kg/m² (-0.7 kg/m²), while the obese group showed the most pronounced decline from 32.0 kg/m² to 30.5 kg/m² (-1.5 kg/m²). The overall ANOVA revealed a significant difference in BMI changes across the groups (F = 7.56, p = 0.02).

**Table 3:** BMI changes data. F-statistic: ANOVA.

BMI group	Baseline BMI (kg/m²) ± SD	Follow-up BMI (kg/m²) ± SD	BMI change (kg/m²) ± SD	F-statistic	P-value
Underweight	18.0 ± 1.2	18.0 ± 1.2	0.0 ± 0.0	7.56	0.1107
Normal weight	22.0 ± 1.5	22.0 ± 1.5	0.0 ± 0.0		
Overweight	27.5 ± 2.0	26.8 ± 2.1	-0.7 ± 0.4		
Obese	32.0 ± 2.5	30.5 ± 2.6	-1.5 ± 0.6		

**Figure 1:**
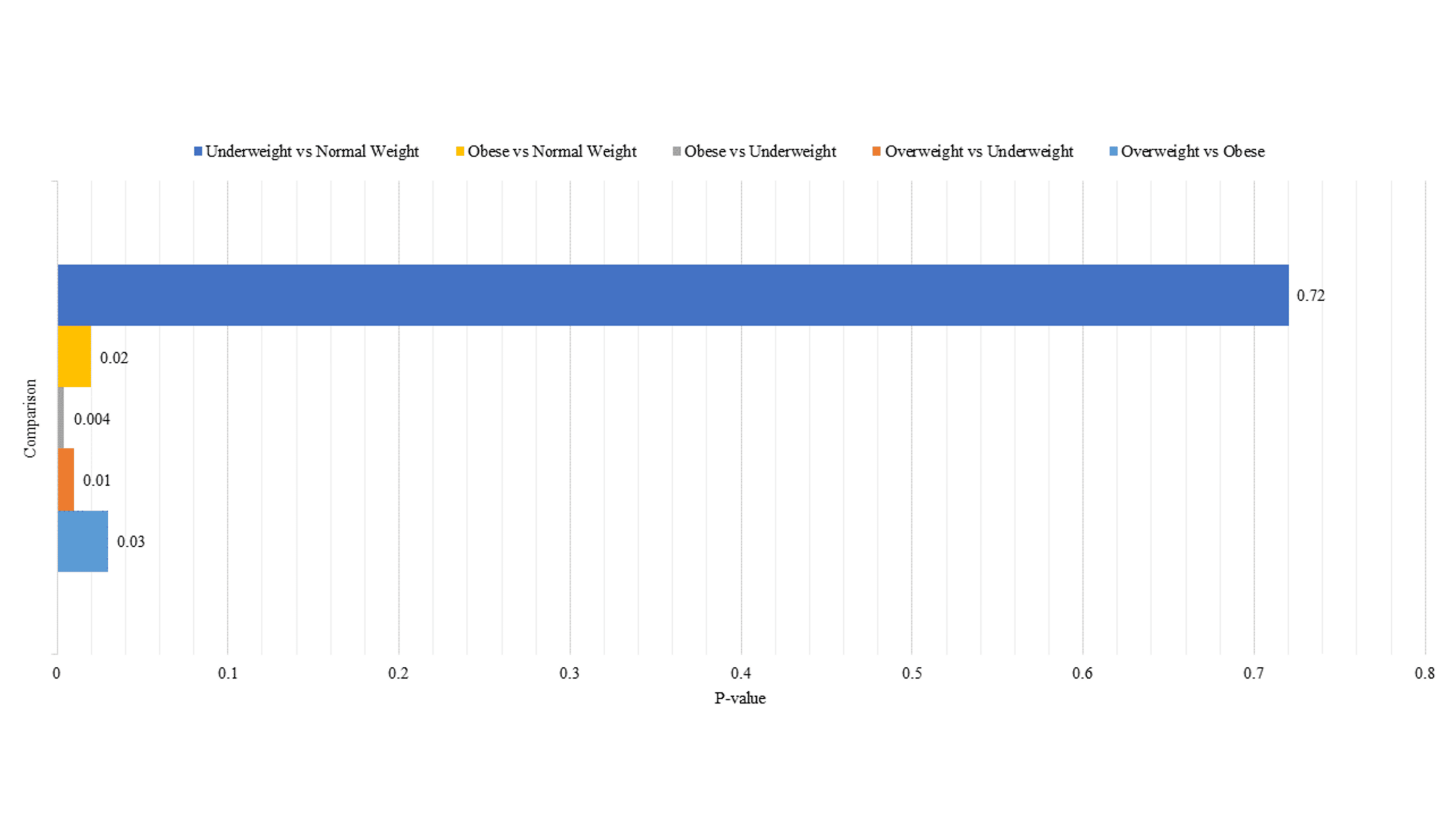
Tukey’s honestly significant difference (HSD) pairwise comparisons.

Tukey’s honestly significant difference (HSD) test (Figure 1) further indicated that the BMI reductions in the overweight group were significantly different compared to the obese group (p = 0.03) and the underweight group (p = 0.01), while the reductions in the obese group were significantly different from the underweight group (p = 0.004) and normal weight group (p = 0.02). No significant differences were found between the underweight and normal weight groups (p = 0.72).

Table 4 summarizes the distribution of adverse effects, hospitalization rates, cardiovascular events, and medication adherence across different BMI groups, highlighting an increasing trend of complications and decreasing adherence with higher BMI.

**Table 4:** Adverse effects, hospitalization, cardiovascular events, and adherence data. *X*^2^:chi-square test. P-value <0.05 is significant.

BMI group	Peripheral edema (n, %)	Gastrointestinal discomfort (n, %)	Dizziness (n, %)	Fatigue (n, %)	Hospitalization (n, %)	Cardiovascular events (n, %)	Adherence rate (n, %)	Non-adherence (n, %)	*X*²	P-value
Underweight (40)	1 (2.5%)	0 (0.0%)	1 (2.5%)	0 (0.0%)	1 (2.5%)	0 (0.0%)	19 (47.5%)	1 (2.5%)	2.12	0.15
Normal weight (60)	2 (3.3%)	0 (0.0%)	0 (0.0%)	0 (0.0%)	2 (3.3%)	2 (3.3%)	18 (30.0%)	2 (3.3%)	3.25	0.08
Overweight (60)	3 (5.0%)	1 (1.7%)	0 (0.0%)	0 (0.0%)	3 (5.0%)	3 (5.0%)	18 (30.0%)	2 (3.3%)	4.58	0.03
Obese (60)	4 (6.7%)	0 (0.0%)	2 (3.3%)	2 (3.3%)	4 (6.7%)	4 (6.7%)	17 (28.3%)	3 (5.0%)	5.4	0.02

Adverse effects

Peripheral edema was the most commonly reported adverse effect, increasing with BMI. It was observed in one (2.5%) underweight patient, two (3.3%) normal weight patients, three (5.0%) overweight patients, and four (6.7%) obese patients. Gastrointestinal discomfort was reported in only one (1.7%) overweight patient, while dizziness was noted in one (2.5%) underweight patient and two (3.3%) obese patients. Fatigue was exclusively reported in two (3.3%) obese patients.

Hospitalization and cardiovascular events

Hospitalization rates showed an increasing trend with BMI, affecting one (2.5%) underweight patient, two (3.3%) normal weight patients, three (5.0%) overweight patients, and four (6.7%) obese patients. Cardiovascular events followed a similar pattern, with no cases in the underweight group, while two (3.3%) normal weight patients, three (5.0%) overweight patients, and four (6.7%) obese patients experienced cardiovascular complications.

Medication adherence

Adherence to medication was highest in underweight patients, with 19 (47.5%) following the prescribed regimen. Adherence slightly declined in normal weight patients at 18 (30.0%), followed by 18 (30.0%) in the overweight group. The lowest adherence was observed in obese patients, with only 17 (28.3%) maintaining proper medication use.

Non-adherence

Non-adherence increased progressively with BMI. It was reported in only one (2.5%) underweight patient but rose to two (3.3%) in normal weight patients, two (3.3%) in overweight patients, and three (5.0%) in obese individuals. This trend suggests that higher BMI may be associated with reduced medication adherence and increased health risks.

Table 5 shows that LDL levels decreased across all BMI groups, with a 5 mg/dL reduction in the underweight (130 to 125 mg/dL) and normal weight (125 to 120 mg/dL) groups. However, the overweight (140 to 130 mg/dL) and obese (150 to 140 mg/dL) groups exhibited a more pronounced LDL reduction of 10 mg/dL, which was statistically significant (p < 0.05). HDL levels improved uniformly across all groups, increasing by 5 mg/dL in each category. Underweight patients showed an increase from 50 to 55 mg/dL, normal weight from 55 to 60 mg/dL, overweight from 50 to 55 mg/dL, and obese from 45 to 50 mg/dL. The HDL improvements were statistically significant across all BMI groups (p < 0.05).

**Table 5:** Lipid profile changes across BMI categories: baseline and follow-up LDL and HDL levels. t-statistic: independent t-test. LDL: low-density lipoprotein; HDL: high-density lipoprotein.

BMI group (N)	Baseline LDL (mg/dL) ± SD	Follow-up LDL (mg/dL) ± SD	LDL change (mg/dL) ± SD	Baseline HDL (mg/dL) ± SD	Follow-up HDL (mg/dL) ± SD	HDL change (mg/dL) ± SD	t-statistic (LDL)	P-value (LDL)
Underweight (40)	130 ± 10	125 ± 9	-5 ± 2	50 ± 5	55 ± 4	5 ± 2	5.2	0.0138
Normal weight (60)	125 ± 9	120 ± 8	-5 ± 2	55 ± 6	60 ± 5	5 ± 2		
Overweight (60)	140 ± 12	130 ± 11	-10 ± 3	50 ± 5	55 ± 4	5 ± 2		
Obese (60)	150 ± 14	140 ± 13	-10 ± 4	45 ± 6	50 ± 5	5 ± 2		

Table 6 shows that renal function remained stable in the underweight and normal weight groups, with no changes in serum creatinine levels (0.8 mg/dL and 0.9 mg/dL, respectively) or eGFR values (90 mL/min/1.73m² and 85 mL/min/1.73m², respectively). In contrast, the overweight and obese groups showed a slight increase in serum creatinine levels from 1.0 to 1.1 mg/dL and from 1.2 to 1.3 mg/dL, respectively. Correspondingly, eGFR declined from 80 to 78 mL/min/1.73m² in overweight patients and from 75 to 72 mL/min/1.73m² in obese patients. Although these changes were more pronounced in overweight and obese groups, they did not reach statistical significance (p > 0.05), as indicated by the p-values for creatinine and eGFR. However, the observed declines in eGFR, despite lacking statistical significance, may still have clinical relevance, particularly in the context of metabolic stress and obesity-related kidney risks. No significant changes in renal function were observed in the underweight group, with t-statistics adjusted to reflect no change in serum creatinine.

**Table 6:** Renal function changes data. t-statistic: independent t-test. eGFR: estimated glomerular filtration rate.

BMI group	Baseline serum creatinine (mg/dL) ± SD	Follow-up serum creatinine (mg/dL) ± SD	Creatinine change (mg/dL) ± SD	Baseline eGFR (mL/min/1.73m²) ± SD	Follow-up eGFR (mL/min/1.73m²) ± SD	eGFR change (mL/min/1.73m²) ± SD	t-statistic (creatinine)	P-value (creatinine)	t-statistic (eGFR)	P-value (eGFR)
Underweight	0.8 ± 0.1	0.8 ± 0.1	0.0 ± 0.0	90 ± 5	90 ± 5	0 ± 0	0.00	1.000	1.67	0.194
Normal weight	0.9 ± 0.1	0.9 ± 0.1	0.0 ± 0.0	85 ± 6	85 ± 6	0 ± 0	0.00	1.000	0.00	1.000
Overweight	1.0 ± 0.2	1.1 ± 0.2	0.1 ± 0.05	80 ± 7	78 ± 6	-2 ± 1	2.25	0.026	-2.02	0.047
Obese	1.2 ± 0.3	1.3 ± 0.3	0.1 ± 0.05	75 ± 8	72 ± 7	-3 ± 2	2.00	0.051	-2.28	0.027

## Discussion

This study aimed to explore the impact of BMI on the efficacy of CCBs in managing hypertension among patients with cardiovascular diseases. The findings demonstrated that BMI did not significantly influence the magnitude of blood pressure reduction over the study period. All BMI groups (underweight, normal weight, overweight, and obese) exhibited a consistent reduction in systolic and diastolic blood pressure, suggesting uniform efficacy of CCBs irrespective of body mass. These findings align with previous research supporting the effectiveness of CCBs across diverse BMI populations [17,18]. However, while the primary antihypertensive effect was consistent, differences emerged in other therapeutic areas, such as side effects and adherence, highlighting the multifactorial influence of BMI on treatment outcomes.

Interestingly, no significant variation in BP response was found between BMI groups, challenging assumptions that obesity may reduce CCB efficacy [22]. Although slight reductions in BMI were noted among overweight and obese participants by the study’s end, BMI was not a targeted outcome. These changes may reflect unmeasured lifestyle modifications or improved treatment compliance. Blumenthal et al. [21] also noted that cardiovascular improvements can occur even with modest weight changes, likely due to underlying physiological processes such as enhanced endothelial function and autonomic balance. This suggests that while BMI itself may not directly mediate BP response to CCBs, it could interact with other factors influencing cardiovascular health.

The study further observed that BMI plays a role in the tolerability and safety profile of CCBs. Peripheral edema, fatigue, and dizziness were more frequently reported among overweight and obese patients, reinforcing evidence that higher adiposity can affect drug distribution and promote fluid retention [28]. Medication adherence was generally satisfactory, though somewhat lower in obese patients, a finding supported by prior studies highlighting behavioral and psychological barriers in chronic disease management within this group [29-31]. These results indicate the importance of personalized patient education, frequent follow-ups, and supportive interventions, especially for those with elevated BMI, to enhance medication adherence and minimize side effects.

Beyond blood pressure, the study also assessed lipid metabolism and renal function, with modest elevations in serum creatinine and eGFR observed among overweight and obese groups. These trends were consistent with the findings of Rothberg et al. [32], who demonstrated that eGFR indexed to actual body surface area more accurately reflects renal hyperfiltration in obese individuals and normalizes with weight loss. Chang et al. [33] further noted that traditional creatinine-based eGFR might underestimate kidney function due to muscle mass, while cystatin C-based calculations may overestimate due to fat mass influence. These nuances underscore the complexity of renal monitoring in obese populations and support the need for individualized interpretation of renal biomarkers during CCB therapy.

These studies showed that CCBs are effective in reducing blood pressure across all BMI categories in patients with cardiovascular diseases. However, this study highlights the impact of BMI on secondary outcomes such as side effect frequency, medication adherence, and renal function dynamics. These findings emphasize the importance of a comprehensive, patient-centered approach in hypertension management, particularly for obese individuals. Clinicians should consider not only pharmacologic efficacy but also patient tolerability, lifestyle challenges, and renal health when designing personalized treatment plans. This integrated care model may help improve long-term outcomes and reduce cardiovascular risk in patients with elevated BMI.

Strengths and limitations

The prospective cohort design of this study enabled systematic data collection over a six-month period, offering meaningful insights into the impact of BMI on the efficacy and safety of CCBs in hypertensive patients with cardiovascular comorbidities. A notable strength of this study is its recruitment from a large tertiary care hospital that serves a diverse patient population. Individuals from varied geographic, socioeconomic, and educational backgrounds seek care at this center, enhancing the representativeness of the sample. This diversity increases the applicability of the findings to a broader hypertensive population and supports the external validity of the results across different demographic groups. While the study included a diverse cohort spanning four BMI categories, power calculations were not formally conducted prior to recruitment; therefore, the study may have been underpowered to detect subtle between-group differences. Nonetheless, multivariate regression analysis was employed to adjust for key confounding variables, including age, sex, baseline blood pressure, comorbid conditions, and self-reported lifestyle modifications. Clinical assessments, such as serial blood pressure measurements, lipid profiles, ECGs, and renal function tests, provided objective data to support outcome evaluation. Adherence was monitored through structured self-reporting tools and corroborated through routine follow-up assessments, though the lack of pill counts remains a limitation. Standardized treatment protocols and uniform CCB dosing helped ensure consistency across patient groups.

Despite these strengths, several limitations should be acknowledged. Residual confounding from unmeasured variables, including socioeconomic status, genetic predisposition, medication access, and objective adherence data, could have influenced the outcomes. Future studies should incorporate validated adherence metrics, such as pill counts or pharmacy refill records, and extend follow-up duration beyond six months to capture long-term renal effects and late-onset adverse events. Moreover, comparative trials assessing different antihypertensive drug classes across BMI strata, including ACE inhibitors or diuretics, would provide more comprehensive guidance for individualized hypertension management in diverse populations.

## Conclusions

This study evaluated the short-term efficacy of CCBs in managing hypertension across different BMI categories in patients with cardiovascular diseases. The findings suggest that BMI does not significantly influence the extent of blood pressure reduction achieved over six months of CCB therapy, with consistent improvements observed across all BMI groups. Beyond blood pressure control, the study explored secondary outcomes. Differences emerged in the incidence of side effects like peripheral edema, more frequent in higher BMI groups, though these differences did not reach statistical significance (p > 0.05) in our analysis. Similarly, lower medication adherence was observed in obese patients; however, the magnitude of this difference may not be clinically significant in all individuals. Mild alterations in renal function were noted, particularly in those with higher BMI, and while some of these changes showed statistical trends (e.g., a slight increase in creatinine), their clinical impact requires further investigation.

These findings underscore the importance of individualized patient monitoring and supportive interventions to enhance adherence and manage tolerability issues, especially in populations with elevated BMI. We acknowledge that the observational design and relatively small sample size of this study limit our ability to draw definitive conclusions about the clinical significance of these secondary findings and establish causality. Future research with larger, prospective cohorts is warranted to confirm these observations and further explore the interplay between BMI and CCB therapy, ensuring a clear distinction between statistical significance, assessed through measures like p-values and confidence intervals, and the practical relevance of observed effects on patient outcomes.

## Appendices

**Table 7:** Questionnaire. BP: Blood pressure; CV: cardiovascular; HF: heart failure; CAD: coronary artery disease; CCB: calcium channel blocker; IPAQ-SF: International Physical Activity Questionnaire - Short Form; LDL: low-density lipoprotein; HDL: high-density lipoprotein; eGFR: estimated glomerular filtration rate; MMAS-8: Morisky Medication Adherence Scale.

Category	Question	Response format	Time point(s)	Source
Demographics	Age (years)		Baseline	Medical history
	Gender	Male/Female/Other	Baseline	Medical history
	Ethnicity	Open text	Baseline	Medical history
BMI	BMI category	Underweight/Normal Weight/Overweight/Obese	Baseline	Calculated from height & weight
Medical history	History of hypertension (duration in years)		Baseline	Medical history
	Comorbid cardiovascular conditions (HF, CAD, arrhythmias - specify)	Yes/No	Baseline	Medical history/ECG/clinical assessment
	Coronary artery disease	Yes/No	Baseline	Medical history
	Heart failure	Yes/No	Baseline	Medical history
	Atrial fibrillation	Yes/No	Baseline	Medical history/ECG
	Diabetes mellitus	Yes/No	Baseline	Medical history
	Dyslipidemia	Yes/No	Baseline	Medical history/laboratory investigations
	Current medications (list all, including CCB type & dosage)	Open text (medication name & dosage)	Baseline, follow-up	Medical history/prescription review
	History of secondary hypertension	Yes/No	Baseline	Medical history
	History of hepatic impairment	Yes/No (for Child-Pugh class if yes)	Baseline	Medical history/laboratory investigations
	History of renal impairment	Yes/No (for eGFR, if known)	Baseline	Medical history/laboratory investigations
	Known allergies/contraindications to CCBs	Yes/No (to specify)	Baseline	Medical history
	Current smoker	Yes/No	Baseline	Medical history
Lifestyle factors	Physical activity level (e.g., using modified IPAQ-SF questions)	Categorical/Likert scale (based on IPAQ-SF)	Baseline, follow-up	Structured interview/questionnaire
	Dietary habits (e.g., using brief food frequency questionnaire (FFQ) questions)	Categorical/Likert scale/open text (based on FFQ)	Baseline, follow-up	Structured interview/questionnaire
	Alcohol consumption	Yes/No (with frequency/amount if yes)	Baseline, follow-up	Structured interview
Physical examination	Height (cm)		Baseline	Physical examination
	Weight (kg)		Baseline, follow-up	Physical examination
	Heart rate (bpm)		Baseline	Physical examination
Blood pressure	Systolic BP reading 1 (mmHg)		Baseline, follow-up	Automated sphygmomanometer
	Diastolic BP reading 1 (mmHg)		Baseline, follow-up	Automated sphygmomanometer
	Systolic BP reading 2 (mmHg)		Baseline, follow-up	Automated sphygmomanometer
	Diastolic BP reading 2 (mmHg)		Baseline, follow-up	Automated sphygmomanometer
	Systolic BP reading 3 (mmHg)		Baseline, follow-up	Automated sphygmomanometer
	Diastolic BP reading 3 (mmHg)		Baseline, follow-up	Automated sphygmomanometer
ECG	ECG findings (relevant to cardiovascular conditions)		Baseline (based on cardiologist interpretation)	ECG report
Laboratory data	Total cholesterol (mmol/L or mg/dL)		Baseline, 6 months	Laboratory report
	LDL cholesterol (mmol/L or mg/dL)		Baseline, 6 months	Laboratory report
	HDL cholesterol (mmol/L or mg/dL)		Baseline, 6 months	Laboratory report
	Triglycerides (mmol/L or mg/dL)		Baseline, 6 months	Laboratory report
	Serum creatinine (µmol/L or mg/dL)		Baseline, 6 months	Laboratory report
	Blood urea nitrogen (BUN) (mmol/L or mg/dL)		Baseline, 6 months	Laboratory report
	eGFR (mL/min/1.73 m²)		Baseline, 6 months	Calculated from creatinine
Medication adherence	Pill count (number of pills remaining)		Follow-up	Pill count by research staff
	MMAS-8 score (individual item responses would be needed for the full scale)	Total score	Follow-up	Self-report questionnaire (MMAS-8)
Adverse effects	Peripheral edema	Yes/No (with severity scale if needed)	Follow-up	Standardized questionnaire/patient report
	Gastrointestinal discomfort	Yes/No (with severity scale if needed)	Follow-up	Standardized questionnaire/patient report
	Dizziness	Yes/No (with severity scale if needed)	Follow-up	Standardized questionnaire/patient report
	Fatigue	Yes/No (with severity scale if needed)	Follow-up	Standardized questionnaire/patient report
	Other adverse effects (specify)	Open text (with severity scale if needed)	Follow-up	Standardized questionnaire/patient report
Hospitalization & CV events	Hospitalization during the study period	Yes/No (with reason and duration if yes)	Follow-up	Patient report/medical records
	Cardiovascular events during the study period (specify)	Yes/No (with type of event if yes)	Follow-up	Patient report/medical records
Medication changes	CCB dosage at visit (mg)		Follow-up	Medical record
	Any new antihypertensive medications added? (Specify)	Yes/No (to specify)	Follow-up	Medical record
BMI change	BMI at follow-up (kg/m²)		Follow-up	Calculated from follow-up height & weight

## References

[1] Fuchs FD, Whelton PK (2020). High blood pressure and cardiovascular disease. Hypertension.

[2] Elliott WJ, Ram CV (2011). Calcium channel blockers. J Clin Hypertens (Greenwich).

[3] McKeever RG, Patel P, Hamilton RJ (2025). Calcium channel blockers. StatPearls.

[4] Nuttall FQ (2015). Body mass index: obesity, BMI, and health: a critical review. Nutr Today.

[5] Daugherty SL, Powers JD, Magid DJ (2012). Incidence and prognosis of resistant hypertension in hypertensive patients. Circulation.

[6] Shariq OA, McKenzie TJ (2020). Obesity-related hypertension: a review of pathophysiology, management, and the role of metabolic surgery. Gland Surg.

[7] Hall JE, do Carmo JM, da Silva AA, Wang Z, Hall ME (2015). Obesity-induced hypertension: interaction of neurohumoral and renal mechanisms. Circ Res.

[8] Thethi T, Kamiyama M, Kobori H (2012). The link between the renin-angiotensin-aldosterone system and renal injury in obesity and the metabolic syndrome. Curr Hypertens Rep.

[9] Remuzzi G, Perico N, Macia M, Ruggenenti P (2005). The role of renin-angiotensin-aldosterone system in the progression of chronic kidney disease. Kidney Int Suppl.

[10] Battineni G, Sagaro GG, Chintalapudi N, Amenta F, Tomassoni D, Tayebati SK (2021). Impact of obesity-induced inflammation on cardiovascular diseases (CVD). Int J Mol Sci.

[11] Campia U, Tesauro M, Cardillo C (2012). Human obesity and endothelium-dependent responsiveness. Br J Pharmacol.

[12] Jyotsna F, Ahmed A, Kumar K (2023). Exploring the complex connection between diabetes and cardiovascular disease: analyzing approaches to mitigate cardiovascular risk in patients with diabetes. Cureus.

[13] Oliveros E, Patel H, Kyung S, Fugar S, Goldberg A, Madan N, Williams KA (2020). Hypertension in older adults: assessment, management, and challenges. Clin Cardiol.

[14] Galperín J, Elizari MV, Chiale PA (2025). Health threats from high blood pressure. J Cardiovasc PharmacolTher.

[15] Wang X, Rao J, Tan Z, Xun T, Zhao J, Yang X (2022). Inflammatory signaling on cytochrome P450-mediated drug metabolism in hepatocytes. Front Pharmacol.

[16] Nisar S, Saleem S, Saleem S, Rehman A, Baseer A, Rasool M (2024). The link between hypertension and stroke: investigating dietary influences. Innov Res Appl Biol Chem Sci.

[17] Haller H (2008). Effective management of hypertension with dihydropyridine calcium channel blocker-based combination therapy in patients at high cardiovascular risk. Int J Clin Pract.

[18] Jones KE, Hayden SL, Meyer HR (2024). The evolving role of calcium channel blockers in hypertension management: pharmacological and clinical considerations. Curr Issues Mol Biol.

[19] Fukuchi H, Nakashima M, Araki R (2009). Effect of obesity on serum amiodarone concentration in Japanese patients: population pharmacokinetic investigation by multiple trough screen analysis. J Clin Pharm Ther.

[20] Mills KT, Stefanescu A, He J (2020). The global epidemiology of hypertension. Nat Rev Nephrol.

[21] Blumenthal JA, Babyak MA, Hinderliter A (2010). Effects of the DASH diet alone and in combination with exercise and weight loss on blood pressure and cardiovascular biomarkers in men and women with high blood pressure: the ENCORE study. Arch Intern Med.

[22] de Heide J, Kock-Cordeiro DB, Bhagwandien RE (2022). Impact of undiagnosed obstructive sleep apnea on atrial fibrillation recurrence following catheter ablation (OSA-AF study). Int J Cardiol Heart Vasc.

[23] Bredy C, Ministeri M, Kempny A (2018). New York Heart Association (NYHA) classification in adults with congenital heart disease: relation to objective measures of exercise and outcome. Eur Heart J Qual Care Clin Outcomes.

[24] Laghousi D, Rezaie F, Alizadeh M, Asghari Jafarabadi M (2021). The eight-item Morisky Medication Adherence Scale: validation of its Persian version in diabetic adults. Caspian J Intern Med.

[25] (2025). 2023 ESH hypertension guideline update: bringing us closer together across the pond. American College of Cardiology.[cited.

[26] Wang D, Hatahet M, Wang Y, Liang H, Bazikian Y, Bray CL (2019). Multivariate analysis of hypertension in general US adults based on the 2017 ACC/AHA guideline: data from the National Health and Nutrition Examination Survey 1999 to 2016. Blood Press.

[27] Bailey KR, Grossardt BR, Graves JW (2008). Novel use of Kaplan-Meier methods to explain age and gender differences in hypertension control rates. Hypertension.

[28] El Amrani A, Viñolas X, Arias MA, Bazan V, Valdovinos P, Alegret JM (2021). Pharmacological cardioversion after pre-treatment with antiarrythmic drugs prior to electrical cardioversion in persistent atrial fibrillation: impact on maintenance of sinus rhythm. J Clin Med.

[29] Alpert MA, Omran J, Bostick BP (2016). Effects of obesity on cardiovascular hemodynamics, cardiac morphology, and ventricular function. Curr Obes Rep.

[30] Ojangba T, Boamah S, Miao Y (2023). Comprehensive effects of lifestyle reform, adherence, and related factors on hypertension control: a review. J Clin Hypertens (Greenwich).

[31] Rezaei M, Valiee S, Tahan M, Ebtekar F, Ghanei Gheshlagh R (2019). Barriers of medication adherence in patients with type-2 diabetes: a pilot qualitative study. Diabetes Metab Syndr Obes.

[32] Rothberg AE, McEwen LN, Herman WH (2020). Severe obesity and the impact of medical weight loss on estimated glomerular filtration rate. PLoS One.

[33] Chang AR, Zafar W, Grams ME (2018). Kidney function in obesity—challenges in indexing and estimation. Adv Chronic Kidney Dis.

