# Correction: Association Between Body Mass Index and the Efficacy of Calcium Channel Blockers for Hypertension in Cardiovascular Disease Patients

**DOI:** 10.7759/cureus.c225

**Published:** 2025-05-22

**Authors:** Bassel Abdallah, Ahmed Jamal Chaudhary, Muhammad Waqas Javed, Marium Nadeem Khan, Ayesha Bibi, Muhammad Fayyaz Zafar, Muhammad Noor, Usman Tariq, Farzana Salman

**Affiliations:** 1 Department of General Internal Medicine, Royal Alexandra Hospital, Glasgow, GBR; 2 Department of Internal Medicine, Detroit Medical Center (DMC) Sinai-Grace Hospital, Michigan State University, Detroit, USA; 3 Department of Medicine, Shifa International Hospital, Shifa College of Medicine, Islamabad, PAK; 4 Department of Cardiology, Mayo Hospital Lahore, Lahore, PAK; 5 Department of Adult Critical Care, Sindh Infectious Diseases Hospital, Karachi, PAK; 6 Department of General Medicine, Countess of Chester Hospital NHS Foundation Trust, Chester, GBR; 7 Department of Internal Medicine, The First Affiliated Hospital of Changsha Medical University, Changsha Medical University, Changsha, CHN; 8 Department of Physiology, Peshawar Medical College, Peshawar, PAK

The institutional affiliation for author Bassel Abdallah has been corrected as follows.

The following affiliations have been removed: 

Department of General Internal Medicine, MD Health Center, Lahore, PAK Department of Medicine, Lahore Medical and Dental College, Lahore, PAK

The following affiliation has been retained: 

Department of General Internal Medicine, Royal Alexandra Hospital, Glasgow, GBR

